# Survival Impact of Chronic Obstructive Pulmonary Disease or Acute Exacerbation on Patients with Rectal Adenocarcinoma Undergoing Curative Resection: A Propensity Score-Matched, Nationwide, Population-Based Cohort Study

**DOI:** 10.3390/cancers13164221

**Published:** 2021-08-22

**Authors:** Jiaqiang Zhang, Kuo-Chin Chiu, Wei-Chun Lin, Szu-Yuan Wu

**Affiliations:** 1 Department of Anesthesiology and Perioperative Medicine, Henan Provincial People’s Hospital, People’s Hospital of Zhengzhou University, Zhengzhou 450052, China; 116096@h.tmu.edu.tw; 2 Division of Chest, Department of Internal Medicine, Lo-Hsu Medical Foundation, Lotung Poh-Ai Hospital, Yilan 256, Taiwan; 979010@mail.pohai.org.tw (K.-C.C.); 973002@mail.pohai.org.tw (W.-C.L.); 3 Department of Food Nutrition and Health Biotechnology, College of Medical and Health Science, Asia University, Taichung 413, Taiwan; 4 Big Data Center, Lo-Hsu Medical Foundation, Lotung Poh-Ai Hospital, Yilan 256, Taiwan; 5 Division of Radiation Oncology, Lo-Hsu Medical Foundation, Lotung Poh-Ai Hospital, Yilan 256, Taiwan; 6 Department of Healthcare Administration, College of Medical and Health Science, Asia University, Taichung 413, Taiwan; 7 Cancer Center, Lo-Hsu Medical Foundation, Lotung Poh-Ai Hospital, Yilan 256, Taiwan; 8 Graduate Institute of Business Administration, Fu Jen Catholic University, Taipei 242062, Taiwan; 9 Centers for Regional Anesthesia and Pain Medicine, Taipei Municipal Wan Fang Hospital, Taipei Medical University, Taipei 110, Taiwan

**Keywords:** rectal adenocarcinoma, COPD, COPDAE, cigarette smoking, survival

## Abstract

**Simple Summary:**

This study is the first to estimate the impact of smoking-related chronic obstructive pulmonary disease (COPD) in patients with rectal adenocarcinoma undergoing curative resection. In these patients, current smokers with smoking-related COPD had worse survival outcomes than nonsmokers without COPD. Moreover, hospitalization for COPD with acute exacerbation within 1 year before diagnosis was an independent risk factor for OS in these patients, with a higher number of hospitalizations being associated with poorer survival.

**Abstract:**

Purpose: The survival effect of current smoking-related chronic obstructive pulmonary disease (COPD) and COPD with acute exacerbation (COPDAE) is unclear for patients with rectal adenocarcinoma undergoing curative resection. Methods: We recruited patients with clinical stage I–IIIC rectal adenocarcinoma from the Taiwan Cancer Registry Database who had received surgery. The Cox proportional hazards model was used to analyze all-cause mortality. We categorized the patients into two groups by using propensity score matching based on COPD status to compare overall survival outcomes: Group 1 (current smokers with COPD) and Group 2 (nonsmokers without COPD). Results: In the multivariate Cox regression analyses, the adjusted hazard ratio (aHR; 95% confidence interval (CI)) of all-cause mortality for Group 1 compared with Group 2 was 1.25 (1.04–1.51). The aHRs (95% cis) of all-cause mortality for frequency of ≥1 hospitalizations for COPDAE or ≥2 hospitalizations within 1 year before diagnosis were 1.17 (1.05–1.51) and 1.48 (1.03–2.41) compared with no COPDAE in patients with rectal adenocarcinoma undergoing curative resection. Conclusion: In patients with rectal adenocarcinoma undergoing curative resection, being a current smoker with COPD (Group 1) was associated with worse survival outcomes than being a nonsmoker without COPD (Group 2). Being hospitalized at least once for COPDAE within 1 year before the diagnosis of rectal adenocarcinoma is an independent risk factor for poor overall survival in these patients, and a higher number of hospitalizations for COPDAE within 1 year before diagnosis was associated with poorer survival.

## 1. Introduction

Cigarette smoking has been associated with increased incidence and mortality of colorectal cancer (CRC) [1]. A meta-analysis of 106 observational studies estimated that, compared with never smokers, the risk of CRC and the mortality risk from CRC were higher among smokers (relative risk (RR) 1.18, 95% confidence interval (CI) 1.11–1.25 and RR 1.25, 95% CI 1.14–1.37, respectively) [2]. For both incidence and mortality, the association was stronger for rectal cancer than colon cancer [1]. For these and many other reasons, smoking should be avoided, especially by CRC survivors [3,4,5,6,7,8]. At least four randomized trials have explored health behavior interventions specifically for CRC survivors [9,10,11,12]. Numerous epidemiologic studies have indicated that smoking is the most critical risk factor for chronic obstructive pulmonary disease (COPD) [13,14,15,16,17,18,19]. In addition, regardless of smoking status, COPD is also an independent risk factor for rectal cancer [20] and is also a strong predictor for intensive care unit admission and mortality after CRC surgery [21,22]; this is as preexisting COPD is an independent risk factor for high-grade complications after treatments [23]. Taken together, both COPD and smoking are independent risk factors or prognostic factors for survival in patients with CRC.

Nevertheless, colon and rectal cancer are different tumor types [24], and a recent consensus has proposed abandoning the designation “CRC” [24]. Anatomically, the risk of rectal cancer is four times higher than that of colon cancer [25], and physical activity helps prevent colon cancer but not rectal cancer [26,27,28]. Clear differences exist in molecular carcinogenesis [29], pathology [30], surgical topography and procedures [31], and multimodal treatments [32,33]. Long-term survival rates in patients with colon cancer are higher than in those with rectal cancer, irrespective of multimodal therapy [24]. Surgery for rectal cancer is associated with higher morbidity and mortality, and with higher local recurrence rates than surgery for colon cancer [24]. Therefore, smoking, COPD, and severity of COPD (COPD with acute exacerbation, COPDAE) may influence overall survival (OS) in patients with rectal cancer as surgery for rectal cancer has higher morbidity and mortality rates than colon cancer [24]. Here, we focused on the impact of the presence and severity of smoking-related COPD on the survival of patients with rectal adenocarcinoma undergoing curative resection.

Data are lacking regarding whether smoking-related COPD or its severity is an independent prognostic factor for OS in patients with rectal adenocarcinoma undergoing curative resection. If these conditions are identified as independent prognostic factors of OS in patients with rectal adenocarcinoma, they can serve as simple and valuable prognostic factors for these patients. Therefore, we conducted a head-to-head propensity score matching (PSM) study to estimate the survival outcomes for patients with smoking-related COPD and nonsmokers without COPD with rectal adenocarcinoma undergoing curative resection. This is the first study to estimate the survival impact of smoking-related COPD on patients with rectal adenocarcinoma undergoing curative resection.

## 2. Patients and Methods

### 2.1. Study Population

The study was a propensity score-matched, nationwide, population-based retrospective cohort study. For this cohort study, we enrolled patients from the Taiwan Cancer Registry Database (TCRD) with a diagnosis of rectal adenocarcinoma between 1 January 2009, and 31 December 2017. The index date was the date of diagnosis, and the follow-up duration was from the index date to 31 December 2019. The TCRD contains detailed cancer-related information of patients, including the pathologic stage, cigarette smoking habit, treatment modalities, pathologic data, irradiation doses, chemotherapy regimen and dosage, margin status, and grade of differentiation [34,35,36,37,38]. The study protocols were reviewed and approved by the Institutional Review Board of Tzu-Chi Medical Foundation (IRB109-015-B). 

### 2.2. Inclusion and Exclusion Criteria 

The diagnoses of the enrolled patients were confirmed after reviewing their pathological data, and the patients with newly diagnosed rectal adenocarcinoma were confirmed to have no other cancers or distant metastases. Rectal adenocarcinoma was defined based on the National Comprehensive Cancer Network (NCCN) guidelines as follows: cancer whose distal border is within 12 cm of the anal verge as determined by rigid proctoscopy [39]. The patients were included if they had received a rectal adenocarcinoma diagnosis and surgery, were ≥20 years old, and had pathologic stages I–IIIC without metastasis, according to the American Joint Committee on Cancer criteria (AJCC, 8th edition). The standard surgery for rectal adenocarcinoma in our study was complete (curative) resection. Neoadjuvant CCRT followed by surgery would be performed for pre-surgery clinical T3 or lymph node positive (AJCC clinical stage II-IIIC) based on NCCN guidelines in Taiwan [39,40,41]. Patients were excluded if they had a history of other cancers before the index date, unknown pathologic types, missing sex data, no smoking habit, unclear pathologic staging, or nonadenocarcinoma histology. In addition, patients with unclear differentiation of tumor grade, missing perineural invasion (PNI) status, missing lymphovascular invasion (LVI) status, or unclear margin status were excluded. The adjuvant treatments of adjuvant radiotherapy (RT), adjuvant chemotherapy, or target therapy were allowed based on NCCN guidelines in Taiwan [39]. Furthermore, we also excluded patients having unclear Charlson comorbidity index (CCI) scores, undergoing curative resection > 3 months after the index date, or having unclear preexisting comorbidities. Smokers were defined as current smokers who had rectal adenocarcinoma receiving surgery. We categorized the enrolled patients into two groups based on their current smoking and COPD status to compare all-cause mortality: Group 1 (current smokers with smoking-related COPD) and Group 2 (never-smokers without COPD). We also estimated the survival outcome associated with the severity of smoking-related COPD (frequency of hospitalization for COPDAE with 0, ≥1, and ≥2 hospitalizations within 1 year before the index date) and of patients with stage I–IIIC rectal adenocarcinoma undergoing curative resection. The incidence of comorbidities was scored using the CCI [42,43]. Diabetes, hyperlipidemia, hypertension, chronic kidney disease (CKD), and cardiovascular diseases were excluded from the CCI scores to prevent repetitive adjustment in multivariate analysis. Only comorbidities observed within 6 months before the index date were included; they were coded and classified according to the International Classification of Diseases, 10th Revision, Clinical Modification (ICD-10-CM) codes at the first admission or after more than two repetitions of a code were issued at outpatient department visits. Supplemental Figure S1 shows the flowchart of patient selection.

### 2.3. Propensity Score Matching and Covariates 

To reduce the effects of potential confounders when all-cause mortality between Groups 1 and 2 were compared, we performed 2:1 PSM with a caliper of 0.2 for the following variables: sex, age, diabetes, hyperlipidemia, hypertension, CKD, cardiovascular diseases, CCI score, pathologic stage, grade of differentiation, LVI, PNI, margin status, income levels, adjuvant chemotherapy, and neoadjuvant concurrent chemoradiotherapy (CCRT) [44]. A Cox proportional hazards model was used to regress all-cause mortality on different COPD statuses, with a robust sandwich estimator used to account for clustering within matched sets [45]. Multivariate Cox regression analyses were performed to calculate hazard ratios (HRs) to determine whether the factors of different COPD status, frequency of hospitalization for COPDAE within 1 year before the index date, age, sex, diabetes, hyperlipidemia, hypertension, CKD, cardiovascular diseases, CCI score, pathologic stage, grade of differentiation, LVI, PNI, margin status, income levels, adjuvant chemotherapy, and neoadjuvant CCRT were potential independent predictors of all-cause mortality. Neoadjuvant CCRT has been considered as a covariate and matching in Table 1. We use pathologic stage as a confounding factor for our patients with rectal adenocarcinoma receiving surgery, as all patients received surgery having pathologic stages. We did not use clinical stages as another covariate, as there is quite strong collinearity between clinical stages and pathologic stages. Positive margin was defended as R1 resection. Potential predictors were controlled for in the PSM (Table 1), and all-cause mortality was the primary endpoint in both groups. We also supplied the characteristics of patients with rectal adenocarcinoma receiving surgery before matching as seen in Supplemental Table S1.

### 2.4. Statistics 

After adjustment for confounders, all analyses were performed using SAS version 9.3 (SAS Institute, Cary, NC, USA). In a two-tailed Wald test, *p* < 0.05 was considered significant. OS was estimated using the Kaplan–Meier method, and differences among the patient categories of non-COPD, COPD, and hospitalization for COPDAE were determined using the stratified log-rank test to compare survival curves (stratified according to matched sets) [46].

## 3. Results

### 3.1. Propensity Score Matching and Study Cohort 

PSM yielded a final cohort of 966 patients with stage I–IIIC rectal adenocarcinoma undergoing curative resection (644 and 322 in Groups 1 and 2, respectively) eligible for further analysis; their characteristics are summarized in Table 1. Age, sex, diabetes, hyperlipidemia, hypertension, CKD, cardiovascular diseases, CCI score, pathologic stage, grade of differentiation, LVI, PNI, margin status, incomes levels, adjuvant chemotherapy, and neoadjuvant CCRT were similar between the two groups due to PSM. Follow-up duration and hospitalization for COPDAE within 1 year before the index date were inconsistent and not matched between the two groups (Table 1). 

### 3.2. Prognostic Factors of All-Cause Mortality after Multivariate Cox Regression Analysis

Multivariate Cox regression analysis indicated that COPD, ≥1 or ≥2 hospitalizations for COPDAE within 1 year before the index date, old age (>65 years old), CCI ≥1, pathologic stage ≥ IIIA, and a moderate–high grade of differentiation were associated with poor OS (Table 2). No significant differences were observed in sex, diabetes, hyperlipidemia, hypertension, CKD, cardiovascular diseases, LVI, PNI, or margin status (Table 2). The adjusted HR (aHR; 95% CI) of all-cause mortality for Group 1 compared with Group 2 was 1.25 (1.04–1.51), *p* = 0.019. The aHRs (95% CIs) of all-cause mortality for ≥1 or ≥2 hospitalizations for COPDAE within 1 year before the index date were 1.17 (1.05–1.51) and 1.48 (1.03–2.41) compared with no COPDAE in patients with rectal adenocarcinoma undergoing curative resection. Moreover, the aHRs (95% CIs) of all-cause mortality for 65–75 years, 75–85 years, and > 85 years, CCI ≥ 1, AJCC pathologic stage IIIA–IIIC, and moderate and high grades of differentiation were 1.37 (1.02–1.84), 2.23 (1.70–3.01), 3.94 (2.61–5.90), 1.49 (1.19–1.86), 1.55 (1.12–2.77), 1.85 (1.18–3.27), 1.97 (1.07–3.58), 1.14 (1.07–1.52), and 1.23 (1.03–1.41), respectively, compared with age ≤ 65 years, CCI = 0, AJCC pathologic stage I, and a low grade of differentiation. 

### 3.3. Kaplan–Meier OS among Non-COPD, COPD, and Hospitalization for COPDAE 

**Figure 1:**
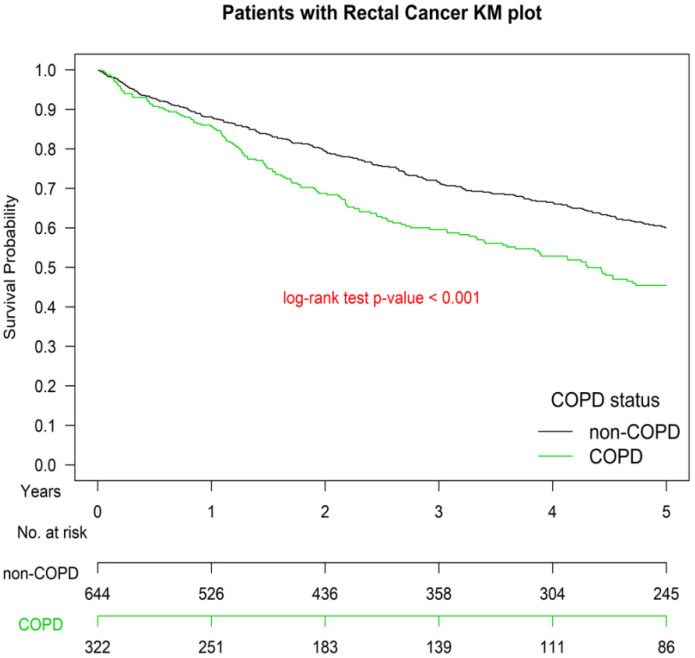
Kaplan–Meier survival curves of patients with rectal adenocarcinoma with or without smoking-related chronic obstructive pulmonary disease before surgery after propensity score matching, COPD, chronic obstruction pulmonary disease.

**Figure 2:**
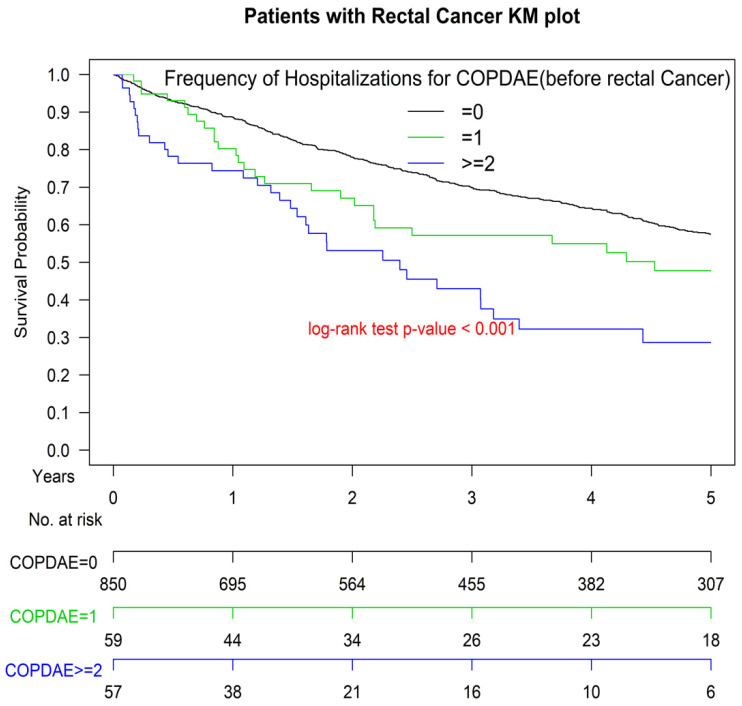
Kaplan–Meier survival curves of patients with rectal adenocarcinoma with hospitalization(s) for acute exacerbations of chronic obstructive pulmonary disease within 1 year before surgery. COPDAE, chronic obstruction pulmonary disease with acute exacerbation.

Figure 1 presents the Kaplan–Meier OS curves for the two groups. The OS of Group 1 was significantly inferior to that of Group 2 (*p* < 0.001). The OS of patients with ≥1 or ≥2 hospitalizations for COPDAE within 1 year before diagnosis was significantly inferior to that of patients with 0 hospitalizations for COPDAE (*p* < 0.001; Figure 2). The worst OS was observed in patients requiring ≥ 2 hospitalizations for COPDAE within 1 year before diagnosis compared with patients with ≥1 or 0 hospitalizations (Figure 2).

## 4. Discussion

Cigarette smoking and COPD are both independent risk factors for rectal adenocarcinoma [1,2,20] However, no report has evaluated the association of preexisting smoking-related COPD, severity of COPD, and survival outcomes among patients with rectal adenocarcinoma undergoing curative resection. Accumulating evidence indicates that smoking might be associated with increased mortality in patients with CRC [9,10,11,12]. Poor prognostic factors of OS in patients with rectal adenocarcinoma include older age [47,48], higher CCI score [49], advanced stage [50], PNI positive [51,52], LVI positive [53], moderate-poor differentiation [54,55,56], margin positive [57,58], low income [59], no adjuvant chemotherapy for advanced stages [60], or no neoadjuvant CCRT use [61]. However, few studies have assessed whether smoking-related COPD or COPDAE within 1 year before the diagnosis of rectal adenocarcinoma is an independent prognostic factor of OS in patients with rectal adenocarcinoma undergoing curative resection. Our study is the first head-to-head PSM design to analyze whether COPD or its severity might be poor prognostic factors in patients with rectal adenocarcinoma undergoing curative resection. Our study may be applied to accentuate the importance of smoking-related COPD management, particularly the identification of frequent exacerbators and the prevention of COPDAE, before rectal adenocarcinoma surgery are initiated.

In Table 1, potential comorbidities or cancer risk factors related with survival in COPD or rectal adenocarcinoma were considered as the covariates through head-to-head PSM and showed a balanced distribution in Groups 1 and 2. COPD has been linked to a number of comorbid conditions [62], such as diabetes, hyperlipidemia, hypertension, CKD, and cardiovascular diseases [63,64,65,66]. Proactive identification and treatment of comorbidities can improve outcomes [67]. Thus, we also considered the aforementioned comorbidities as covariates through the PSM design to balance distribution between the two groups (Table 1). Due to the head-to-head PSM design, all covariates were balanced, and all-cause mortality was significantly higher in Group 1 than in Group 2. 

Multivariate Cox regression analysis indicated that COPD and ≥1 or ≥2 hospitalizations for COPDAE within 1 year before the index date were poor prognostic factors of OS (Table 2). Our study is the first study to estimate the survival impact of COPD (Figure 1) and its severity (measured as number of hospitalizations) before the diagnosis of rectal adenocarcinoma in patients with rectal adenocarcinoma (Figure 2). The reason for this association may be that COPD, especially severe COPD, can increase the risk of more intolerable cardiotoxicity or treatment-related toxicity, decrease the cancer-treatment completion rate, or cause more major complications after treatment [21,22,23,68,69]. Another possible explanation is that smoking may cause more aggressive rectal adenocarcinoma [2], and active and heavy smoking is more common among COPDAE phenotypes [70]. Therefore, smoking-related COPD and COPDAE might be alternative preoperative markers of survival in patients undergoing curative resection for rectal adenocarcinoma. Furthermore, a higher number of hospitalizations for COPDAE within 1 year before the index date was associated with worse survival (Table 2 and Figure 2). Future clinical trials should verify whether these novel prognostic factors of OS for rectal adenocarcinoma identified in the current study affect survival rates. Moreover, in a future study, we aim to evaluate the duration of smoking cessation that may be effective for decreasing all-cause mortality in patients with rectal adenocarcinoma undergoing curative resection. 

In addition, data from randomized trials and a meta-analysis [61] have suggested that the preoperative approach is associated with a more favorable long-term toxicity profile and fewer local recurrences than postoperative therapy; OS appears to be similar. Our findings for neoadjuvant CCRT also indicated similar OS outcomes between neoadjuvant CCRT or non-neoadjuvant CCRT after multivariate analysis (Table 2) [61]. 

The strength of our study was that it was the first and largest cohort study to estimate the survival outcomes of current smokers with smoking-related COPD compared with nonsmokers without COPD in patients with rectal adenocarcinoma receiving curative-intent treatments based on NCCN guidelines [39]. PSM led to comparable covariates between groups, and no selection bias was noted. No study has estimated the impact of COPD and hospitalization for COPDAE in patients with rectal adenocarcinoma undergoing curative resection, and all prognostic factors were evaluated. 

This study has some limitations. First, all patients with rectal adenocarcinoma were enrolled from an Asian population; hence, our results should be cautiously extrapolated to non-Asian populations. However, no evidence has indicated differences in oncologic outcomes for rectal adenocarcinoma undergoing curative resection between Asian and non-Asian populations. Second, the diagnoses of all comorbid conditions were based on ICD-10-CM codes. The Taiwan Cancer Registry Administration randomly reviews charts and interviews patients to verify the accuracy of the diagnoses, and hospitals with outlier charges or practices are audited and heavily penalized if malpractice or discrepancies are identified. Nevertheless, to obtain crucial information on population specificity and disease occurrence, a large-scale randomized trial comparing carefully selected patients undergoing suitable treatments is essential. Third, there is a risk of bias in the study by limiting the cohort to surgical patients, as those patients did not reach surgery due to comorbidities such as COPD. However, the patients did not reach surgery due to multiple comorbidities such as cardiovascular diseases, dementia, poor self-care, or other severe comorbidities, not only COPD. In the current database, we cannot clarify the reasons that the patients did not reach surgery. These potential biases might exist in the study. Nevertheless, being hospitalized at least once for COPDAE within 1 year before the surgery of rectal adenocarcinoma is an independent risk factor for poor overall survival in these patients, and a higher number of hospitalizations for COPDAE within 1 year before diagnosis was associated with poorer survival. The conclusion would not be turned over in the current study. Fourth, in order to diminish the potential bias of patients with rectal adenocarcinoma failing to receive standard of care treatment for rectal cancer, we have considered the therapeutic covariates such as neoadjuvant CCRT, adjuvant chemotherapy use, different pathologic stages, margin status, PNI, LVI, income levels, and adjuvant chemotherapy into the analysis. After PSM adjustment, we believe treatments were similar between the two groups. Finally, the TCRD does not contain information on dietary habits, socioeconomic status, or body mass index, all of which may be risk factors for mortality in patients with rectal adenocarcinoma. However, considering the magnitude and statistical significance of the observed effects in the current study, these limitations are unlikely to affect the conclusions.

## 5. Conclusions

Among patients with rectal adenocarcinoma undergoing curative resection, current smokers with smoking-related COPD had worse survival outcomes than nonsmokers without COPD. Hospitalization for COPDAE within 1 year before diagnosis was found to be an independent risk factor for OS in these patients, with a higher number of hospitalizations being associated with poorer survival.

## Figures and Tables

**Table 1:** Characteristics of patients with rectal adenocarcinoma with or without smoking-related chronic obstructive pulmonary disease before surgery after propensity score matching.

Variables	Non-COPD Patients		COPD Patients		*p*-Value
	*N* = 644	(100%)	*N* = 322	(100%)	
Age (mean ± SD)	(71.96 ± 10.10)		(71.51 ± 9.72)		0.821
Age (years)					0.372
≤65	153	23.76%	74	22.98%	
65–75	224	34.78%	109	33.85%	
75–85	221	34.32%	110	34.16%	
>85	46	7.14%	29	9.00%	
Sex					0.788
Female	205	31.83%	99	30.75%	
Male	439	68.17%	223	69.25%	
Diabetes					0.306
No	462	71.74%	220	68.32%	
Yes	182	28.26%	102	31.68%	
Hyperlipidemia					0.588
No	470	72.98%	241	74.84%	
Yes	174	27.02%	81	25.16%	
Hypertension					0.769
No	587	91.15%	292	90.68%	
Yes	57	8.85%	30	9.32%	
CKD					0.998
No	633	98.29%	316	98.14%	
Yes	11	1.71%	6	1.86%	
Cardiovascular diseases					0.822
No	551	85.56%	273	84.78%	
Yes	93	14.44%	49	15.22%	
CCI score					0.876
0	394	61.18%	200	62.11%	
≥1	250	38.82%	122	37.89%	
AJCC pathologic stages					0.950
I	27	4.19%	16	4.97%	
II	180	27.95%	90	27.95%	
IIIA	177	27.48%	87	27.02%	
IIIB	216	33.54%	106	32.92%	
IIIC	44	6.83%	23	7.14%	
Grade of differentiation					0.881
Low	201	31.21%	107	33.23%	
Moderate	315	48.91%	154	47.83%	
High	128	19.88%	61	18.94%	
Lymphovascular invasion					0.726
No	401	62.27%	196	60.87%	
Yes	243	37.73%	126	39.13%	
Perineural invasion					0.741
No	440	68.32%	223	69.25%	
Yes	204	31.68%	99	30.75%	
Margin (distal and circumferential margin)					0.212
Negative	619	96.12%	308	95.65%	
Positive	25	3.88%	14	4.35%	
Neoadjuvant CCRT					0.601
No	307	47.67%	160	49.69%	
Yes	337	52.33%	162	50.31%	
Adjuvant chemotherapy					0.855
No	295	45.81%	149	46.63%	
Yes	349	54.19%	173	53.73%	
Incomes Levels					0.811
Low (<20,000 NTD)	330	51.24%	168	52.17%	
Middle (20,000–30,000 NTD)	229	35.56%	116	36.02%	
High (>30,000 NTD)	85	13.20%	38	11.81%	
Frequency of Hospitalizations for COPDAE (1 year before rectal cancer)					<0.001
0	644	100.00%	206	63.98%	
1	0	0.00%	59	18.32%	
≥2	0	0.00%	57	17.70%	
Follow up (Death)					<0.001
Years, Median (IQR, Q1, Q3)	4.64 (1.53, 7.04)		3.40 (1.11, 5.53)		
Follow up (Death)					<0.001
Years (mean ± SD)	5.74 ± 4.06		3.95 ± 3.45		
Death					0.039
No	346	53.73%	148	45.96%	
Yes	298	46.27%	174	54.04%	

IQR, interquartile range; SD, standard deviation; AJCC, American Joint Committee on Cancer; CCI, Charlson comorbidity index; COPD, chronic obstructive pulmonary disease; COPDAE, COPD with acute exacerbation; CCRT, concurrent chemoradiotherapy; CKD, chronic kidney disease; and NTD, New Taiwan Dollar.

**Table 2:** Cox proportional hazards models of all-cause mortality for patients with rectal adenocarcinoma with or without smoking-related chronic obstructive pulmonary disease before surgery.

Variables	Crude HR (95% CI)		Adjusted HR * (95% CI)		*p*-Value
COPD status (ref: non-COPD)					
COPD	1.41	(1.17, 1.71)	1.25	(1.04, 1.51)	0.019
Frequency of hospitalization for COPDAE before rectal cancer (ref: 0)					
1	1.42	(1.01, 1.93)	1.17	(1.05, 1.51)	0.021
≥2	2.33	(1.63, 3.36)	1.48	(1.03, 2.41)	0.024
Sex (ref: Female)					
Male	0.95	(0.79, 1.16)	1.09	(0.87, 1.31)	0.432
Age (years; ref: ≤65 y)					
65–75	1.71	(1.27, 2.25)	1.37	(1.02, 1.84)	0.023
75–85	2.44	(1.75, 3.33)	2.23	(1.70, 3.01)	<0.001
>85	4.62	(3.30, 6.91)	3.94	(2.61, 5.90)	<0.001
CCI Score (ref: 0)					
≥1	1.61	(1.34, 1.93)	1.49	(1.19, 1.86)	<0.001
Diabetes (ref: No)					
Yes	1.46	(1.15, 1.86)	1.16	(0.86, 1.57)	0.284
Hyperlipidemia (ref: No)					
Yes	1.02	(0.73, 1.13)	1.01	(0.63, 1.04)	0.183
Hypertension (ref: No)					
Yes	1.07	(0.78, 1.19)	1.01	(0.71, 1.14)	0.691
Cardiovascular diseases (ref: No)					
Yes	1.91	(1.21, 2.44)	1.17	(0.88, 1.81)	0.277
CKD (ref: No)					
Yes	1.07	(0.80, 1.19)	1.04	(0.73, 1.16)	0.505
Lymphovascular invasion (ref: No)					
Yes	1.41	(1.10, 1.81)	1.12	(0.81, 1.57)	0.288
Perineural invasion (ref: No)					
Yes	1.82	(1.36, 2.42)	1.16	(0.78, 1.69)	0.295
Pathologic stage (ref: Stage I)					
II	1.72	(1.02, 3.11)	1.21	(0.81, 2.19)	0.462
IIIA	2.13	(1.55, 2.90)	1.55	(1.12, 2.77)	0.021
IIIB	2.85	(1.70, 3.71)	1.85	(1.18, 3.27)	0.019
IIIC	3.26	(1.80, 6.19)	1.97	(1.07, 3.58)	0.031
Grade of differentiation (ref: Low grade)					
Moderate	1.37	(1.12, 1.68)	1.14	(1.07, 1.52)	0.009
High	1.44	(1.18, 1.71)	1.23	(1.03, 1.41)	0.017
Neoadjuvant CCRT (ref: No)					
Yes	0.71	(0.60, 1.04)	0.81	(0.63, 1.03)	0.213
Adjuvant chemotherapy (ref: No)					
Yes	0.88	(0.75, 1.19)	0.86	(0.73, 1.15)	0.534
Incomes Levels (ref: low)					
Middle (20,000–30,000 NTD)	1.11	(1.01, 1.27)	1.03	(0.97, 1.21)	0.059
High (>30,000 NTD)	1.24	(1.03, 1.39)	1.13	(0.83, 1.30)	0.066
Margin status (ref: Negative)					
Positive	1.09	(0.91, 1.32)	1.04	(0.90, 1.30)	0.057

aHR, adjusted hazard ratio; CIs, confidence intervals; AJCC, American Joint Committee on Cancer; CCI, Charlson comorbidity index; COPD, chronic obstructive pulmonary disease; COPDAE, COPD with acute exacerbation; CCRT, concurrent chemoradiotherapy; CKD, chronic kidney disease; and NTD, New Taiwan Dollar. * All covariates mentioned in Table 2 were adjusted.

## Data Availability

The data sets supporting the study conclusions are included in this manuscript and its supplementary Files.

## Supplementary Materials

The following are available online at https://www.mdpi.com/article/10.3390/cancers13164221/s1, Figure S1: Flow-chart of patient selection, Table S1: Characteristics of patients with rectal adenocarcinoma with or without smoking-related chronic obstructive pulmonary disease before surgery before propensity score matching.

## Abbreviations

COPD: chronic obstructive pulmonary disease; COPDAE, COPD with acute exacerbation; aHR, adjusted hazard ratio; CI, confidence interval; AJCC, American Joint Committee on Cancer; TCRD, Taiwan Cancer Registry Database; PSM, propensity score matching; SD, standard deviation; OS, overall survival; CKD, chronic kidney disease; CCI, Charlson comorbidity index; ICD-10-CM, *International Classification of Diseases, 10th Revision, Clinical Modification*; NCCN, National Comprehensive Cancer Network; CRC, colorectal cancer; RR, relative risk; OS, overall survival; PSM, propensity score matching; PNI, perineural invasion; LVI, lymphovascular invasion; RT, radiotherapy; and CCRT, concurrent chemoradiotherapy
