# Supplementary material for: Survival Impact of Chronic Obstructive Pulmonary Disease or Acute Exacerbation on Patients with Rectal Adenocarcinoma Undergoing Curative Resection: A Propensity Score-Matched, Nationwide, Population-Based Cohort Study
Source: Cancers (Basel). 2021 Aug 22;13(16):4221. doi: 10.3390/cancers13164221 (PMC8391389; doi:10.3390/cancers13164221)
Supplement: Supplementary file 1 [file cancers-13-04221-s001.zip › cancers-1288138-supplementary.pdf]

Table S1. Characteristics of patients with rectal adenocarcinoma with or without smoking-related chronic obstructive pulmonary disease before surgery before propensity score matching.

	**Non-COPD patients**		COPD patients		P-value
	**N = 2,261**	(100%)	N = 322	(100%)	
Age (mean ± SD)	(69.26±13.12)		(71.51 ± 9.72)		<0.001
Age (years)					<0.001
≤65	1,213	53.65%	74	22.98%	
65–75	593	26.23%	109	33.85%	
75–85	379	16.76%	110	34.16%	
>85	76	3.36%	29	9.00%	
Sex					<0.001
Female	952	42.11%	99	30.75%	
Male	1,309	57.89%	223	69.25%	
Diabetes					<0.001
No	1,816	80.32%	220	68.32%	
Yes	445	19.68%	102	31.68%	
Hyperlipidemia					0.073
No	1,795	79.39%	241	74.84%	
Yes	466	20.61%	81	25.16%	
Hypertension					0.069
No	2,125	93.98%	292	90.68%	
Yes	136	6.02%	30	9.32%	
CKD					0.095
No	2,244	99.25%	316	98.14%	
Yes	17	0.75%	6	1.86%	
Cardiovascular diseases					<0.001
No	2,128	94.12%	273	84.78%	
Yes	133	5.88%	49	15.22%	
CCI score					<0.001
0	1,978	87.48%	200	62.11%	
≥1	283	12.52%	122	37.89%	
AJCC pathologic stages					0.809
I	111	4.91%	16	4.97%	
II	626	27.69%	90	27.95%	
IIIA	621	27.47%	87	27.02%	
IIIB	746	33.99%	106	32.92%	
IIIC	157	6.94%	23	7.14%	
Grade of differentiation					0.087
Low	701	31.01%	107	33.23%	
Moderate	1,105	48.87%	154	47.83%	
High	455	20.12%	61	18.94%	
Lymphovascular invasion					0.071
No	1,425	63.01%	196	60.87%	
Yes	836	36.99%	126	39.13%	
Perineural invasion					0.677
No	1,538	68.02%	223	69.25%	
Yes	723	31.98%	99	30.75%	
Margin (distal and circumferential margin)					0.112
Negative	2,176	96.24%	308	95.65%	
Positive	85	3.76%	14	4.35%	
Neoadjuvant CCRT					0.677
No	1,086	48.03%	160	49.69%	
Yes	1,175	51.97%	162	50.31%	
Adjuvant chemotherapy					0.346
No	1,018	45.02%	149	46.63%	
Yes	1,243	54.98%	173	53.73%	

Incomes Levels					<0.001
Low (< 20000 NTD$)	1,198	52.99%	168	52.17%	
Middle (20000-30000 NTD$)	565	24.99%	116	36.02%	
High (>30000 NTD$)	498	22.02%	38	11.81%	
Frequency of Hospitalizations for COPDAE (1 year before rectal cancer)					<0.001
0	2,261	100.00%	206	63.98%	
1	0	0.00%	59	18.32%	
≥2	0	0.00%	57	17.70%	
Follow up (Death)					<0.001
Years, Median (IQR, Q1, Q3)	4.43 (1.74, 6.14)		3.40 (1.11, 5.53)		
Follow up (Death)					<0.001
Years, (mean ± SD)	4.82 ± 3.85		3.95 ± 3.45		
Death					0.013
No	1,175	51.97%	148	45.96%	
Yes	1,086	48.03%	174	54.04%	

IQR, interquartile range; SD, standard deviation; AJCC, American Joint Committee on Cancer; CCI, Charlson comorbidity index; COPD, chronic obstructive pulmonary disease; COPDAE, COPD with acute exacerbation; CCRT, concurrent chemoradiotherapy; CKD, chronic kidney disease; NTD$, New Taiwan Dollar.

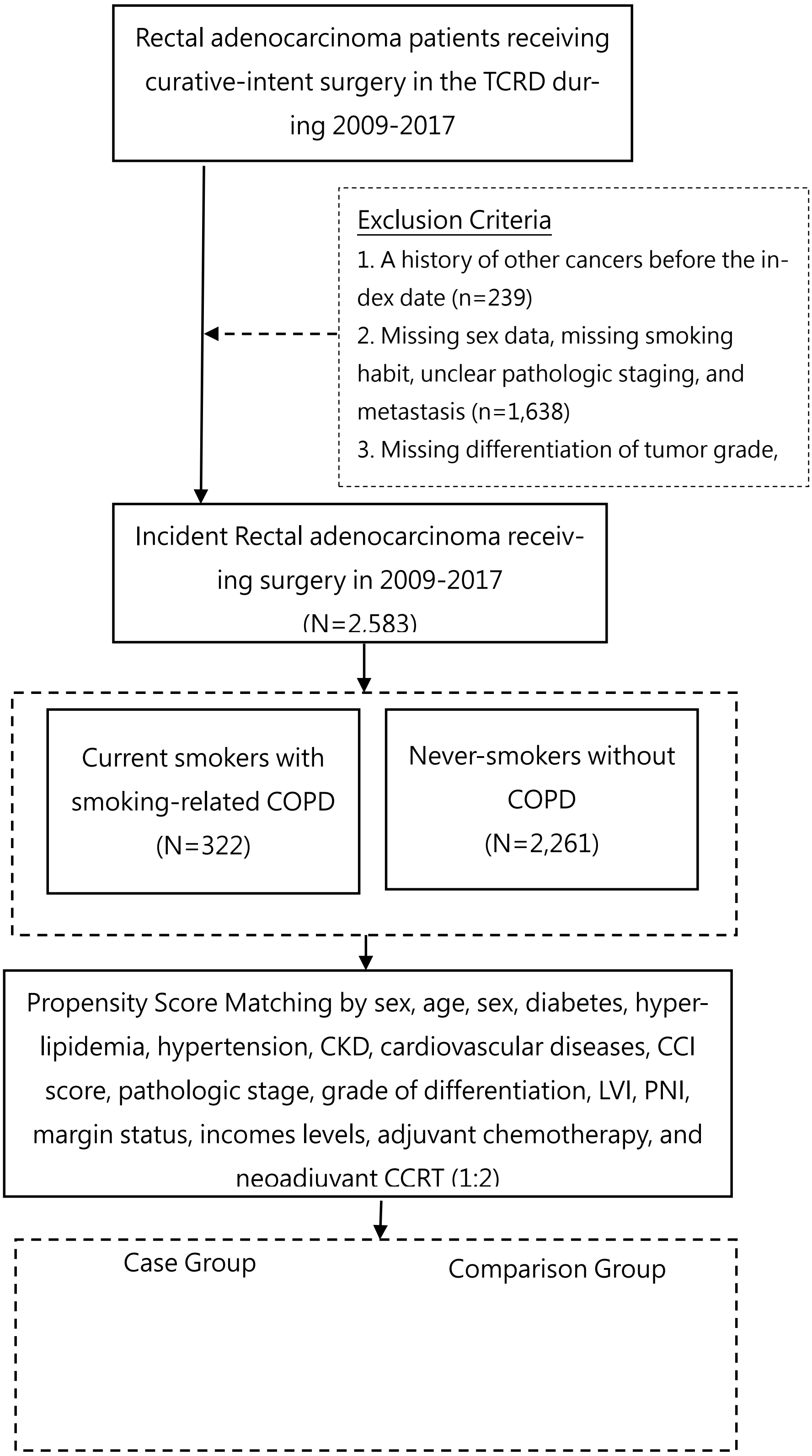

Figure S1. Flow-chart of patient selection.
